# Efficacy of Controlled Atmosphere Treatments to Manage Arthropod Pests of Dry-Cured Hams

**DOI:** 10.3390/insects7030044

**Published:** 2016-09-02

**Authors:** Md. Mahbub Hasan, Michael J. Aikins, Wes Schilling, Thomas W. Phillips

**Affiliations:** 1 Department of Entomology, Kansas State University, 123W. Waters Hall, Manhattan, KS 66506, USA; mmhbgd@yahoo.com (M.M.H.); mja8338@ksu.edu (M.J.A.); 2 Department of Zoology, Rajshahi University, Rajshahi 6205, Bangladesh; 3 Department of Food Science, Nutrition and Health Promotion, Mississippi State University, Starkville, MS 39762, USA; schilling@foodscience.msstate.edu

**Keywords:** ozone, carbon dioxide, low oxygen, low pressure, red-legged ham beetle, *Necrobia rufipes*, ham mite, mold mite, cheese mite, *Tyrophagus putrescentiae*

## Abstract

Research here explored the use of controlled atmospheres (CA) for managing arthropod pests that infest dry-cured hams. Experiments were conducted with low oxygen (O_2_) achieved with low pressure under a vacuum, high carbon dioxide (CO_2_), and ozone (O_3_). Results showed that both low O_2_ and high CO_2_ levels required exposures up to 144 h to kill 100% of all stages of red-legged ham beetle, *Necrobia rufipes* (De Geer) (Coleoptera: Cleridae) and ham mite *Tyrophagus putrescentiae* (Schrank) (Sarcoptiformes: Acaridae) at 23 °C. In addition, both low O_2_ and high CO_2_ had no significant mortality against the ham beetle and ham mites at short exposures ranging from 12 to 48 h. Ham beetles were more tolerant than ham mites to an atmosphere of 75.1% CO_2_ and low pressure of 25 mm Hg, which imposed an atmosphere estimated at 0.9% O_2_. Both low O_2_ and high CO_2_ trials indicated that the egg stages of both species were more tolerant than other stages tested, but *N. rufipes* eggs and pupae were more susceptible than larvae and adults to high concentration ozone treatments. The results indicate that O_3_ has potential to control ham beetles and ham mites, particularly at ≈166 ppm in just a 24 h exposure period, but O_3_ is known from other work to have poor penetration ability, thus it may be more difficult to apply effectively than low O_2_ or high CO_2_. would be. CA treatment for arthropod pests of dry-cured hams show promise as components of integrated pest management programs after methyl bromide is no longer available for use.

## 1. Introduction

Chemical pesticides have been the predominant tools used to control infestations of arthropods, microbes, or vertebrates in durable food commodities and their storage structures over the past several decades. An increased awareness by environmental regulators, health agencies, and consumers of the potential harm from chemical residues in food and effects on the environment led to the restricted use of chemical pesticides in and around food. Chemical fumigation with methyl bromide (MB) was a very common method used for controlling arthropod pests in durable food products, but MB fumigation has been phased out from most general usage in many countries at this writing, and is in the process of being phased out worldwide due to its chemical nature as an ozone-depleting substance that can diminish the protective ozone layer of Earth’s atmosphere [1,2]. The ban on MB has stimulated research on MB alternatives, both chemical and non-chemical approaches, over the past two decades during the recent MB phase-out period [3]. Alternative fumigants, such as hydrogen phosphide, known as phosphine, and sulfuryl fluoride, have been investigated as replacements for MB, and sulfuryl fluoride became registered for use on stored products in many countries [4]. Other gasses that can act as fumigants, as well as non-chemical physical controls for stored-product insects, have also received research attention [2,3].

Insecticidal controlled atmosphere (CA) treatments of commodities and their storage structures represent nonchemical alternatives to fumigants [5,6,7]. Application of CA involves making an artificial change in the composition of atmospheric gases to yield an atmosphere that causes mortality of aerobic organisms. Therefore, the artificial decrease in O_2_ or increase of CO_2_ in a chamber, room or building with an infested commodity, and the active maintenance of such atmospheric changes, is considered a CA treatment [6]. CA differs from “modified atmospheres”, in which the atmospheric gases undergo a slow biologically-based change in percent composition, such that O_2_ decreases while CO_2_ increases over several days or weeks due to the application of a hermetic seal over the commodity or its storage structure. Such a change in natural gas levels during hermetic storage is thought to be due to constricted or altered respiration of microbes, insects, and plant material [5,8,9]. CA treatments with reduced O_2_ and elevated CO_2_ atmospheres have been used for many years to control stored product pests in grains [10,11] and such treatments can be done over short periods, similar to times needed for chemical fumigation. The effectiveness of CA for the control of various insect and mite pests has been tested in the laboratory and under industrial conditions [9,12,13]. Carbon dioxide is toxic to insect pests because it reduces oxygen metabolism below that needed to support life. Data on the effects of different types of CO_2_ treatments and dosages on key pests are available for many species and stages of stored product pests under particular sets of conditions [14]. Depending on the temperature, CO_2_ treatments may take from a few days to several weeks to be effective in gas-tight chambers or silos [15]. CA has distinct biological effects on insects, and these effects can be both physiological [15,16] and behavioral [17]. A CA of low oxygen can be achieved either by purging the storage containers with an inert gas, such as nitrogen, which forces most other gases out and, thus, decreases O_2_ concentration, or it can be achieved by imposing a low pressure via vacuum, which reduces concentrations of all gases in the system [18]. Ozone, O_3_, is a triatomic allotrope of oxygen that occurs in the atmosphere at a very low concentration of 0.6 ppm, but that can be industrially generated for purposes of sterilization and sanitation, such as in public water purification. O_3_ has been investigated for pest control in stored grain with some success and it shows potential for application [19].

This article describes research on CAs for controlling the two most serious arthropod pests of dry-cured hams, the ham mite, also known as the mold mite or cheese mite, *Tyrophagus putrescentiae* (Schrank) (Sarcoptiformes: Acaridae) and the red-legged ham beetle, *Necrobia rufipes* (De Geer) (Coleoptera: Cleridae). Dry-cured ham, also called southern dry cured ham in the USA and similar to many dried meats in other countries, is a pork product from the hind femur that is preserved and dehydrated with heavy application of salt soon after butchering, sometimes followed by smoking for additional characteristic flavoring, and then is aged at room temperature for up to two years [20]. Dry-cured hams, as with other dried meats and aged cheese, can be stored without refrigeration and are susceptible to several species of stored-product pests [21,22]. *T. putrescentiae* is an insidious pest with a very high rate of population increase that has been established as a serious pest of pet foods and numerous dried meats and cheeses [23,24]. The red-legged ham beetle is known for its ability to consume dried meats from bones of vertebrate carrion and, together with the ham mite and several other peri-domestic pests, is part of the pest complex challenging the dry-cured ham industry [25]. MB fumigation has been the predominant and singular method for pest mitigation in dry-cured hams, with no other practices besides sanitation and inspection to address or prevent mite and ham beetle infestations [26]. The ban on MB in the USA has allowed only limited use of specially approved or existing stocks of the commercial fumigant, which will be totally exhausted in the future, and has stimulated research into MB alternatives. Although work to date on MB alternatives for ham pests has investigated several chemical and non-chemical alternatives, none have investigated the potential for CA to control these pests. The purpose of the current study was to evaluate the toxicity of reduced O_2_, increased CO_2_, and application of generated O_3_ against *N. rufipes* and *T. putrescentiae* under laboratory conditions as alternatives to MB.

## 2. Materials and Methods

### 2.1. Mite and Beetle Cultures

*T. putrescentiae* and *N. rufipes* used in the present investigation were from cultures maintained at the Department of Entomology, Kansas State University, USA beginning in 2008 and both originating from active infestations. The laboratory diet for *T. putrescentiae* was the same as that used in our recent work [27] and was composed mostly of water, ground pet food, wheat germ, brewer’s yeast, a vitamin mix, agar, glycerol, and methyl-p-benzoate as a preservative. *N. rufipes* was reared on mixed foods of dried fish, pet food, and ham pieces as described in detail by Hasan and Phillips [28]. Both cultures were maintained in an incubator at 27 °C and 70% RH with a photoperiod of 16:8 h, L:D.

### 2.2. Preparation and Evaluation of Bioassays

All CA applications to mites and beetles were conducted using thick-walled glass Erlenmeyer flasks, 1.0 L with a side-arm port and a neck opening at the top that received an appropriate-sized rubber stopper (Fisher Scientific, Buffalo, NY, USA). A given flask that contained mites and/or beetles destined for exposure to a given treatment represented one replicate for that treatment in a given experiment. All bioassay animals were confined as small groups in 18 mL glass shell vials (Fisherbrand^®^, Buffalo, NY, USA) fitted with a ventilated cap. To determine the efficacy of CAs to control *N. rufipes*, bioassay vials containing separate life stages were placed separately in an exposure flask. The following age life stages of *N. rufipes* were prepared for bioassay: adults that were 7–10 days post-eclosion; 2–3 days old pupae; mature larvae 30–35 days old; and eggs 1–2 days old. Eggs of *N. rufipes* were harvested daily from small groups of adults in small glass jars for which the diet was changed daily. Late larvae were harvested by size directly from the general colony while pupae and adults were generated by holding single individuals of the preceding life stage in single ventilated vials. Ten of a single stage of *N. rufipes* were placed together in a shell vial with 0.5 g of rearing diet and three replicates of each life stage (in three separate exposure flasks) were prepared for a given experiment. Three replicates of untreated reference samples were kept for each life stage as untreated experimental controls. Eggs of *T. putrescentiae* were harvested daily from ventilated vials containing a small amount of diet and 20 or more mixed-sex adults using a single-hair brush. Nymphs and mixed-sex adults of *T. putrescentiae*, hereafter referred to as “mobile stages”, were harvested daily from low-density mite cultures and 20 of these mites were placed in single exposure vials with a small amount of food just before testing. CA exposures to CO_2_ and low O_2_ were conducted in an incubator (Thermo Scientific, Buffalo, NY, USA) set at 23 °C and each flask had a 65%–85% RH achieved by adding water to each before sealing. These are the conditions most common in many dry-cured ham aging rooms [29]. The ozone treatments were applied under ambient laboratory conditions, approximately 27 °C and 40% RH, in a fume hood for worker protection as the application required that O_3_ to be introduced and exited from each flask as a constant flow-through of gas at the desired concentration in air. A flow-through system was needed for O_3_ because a closed system, like we used for CO_2_ and low O_2_, would have an immediate breakdown of O_3_ to O_2_ and ionic O^−^ from the reaction with the glass surfaces, thus resulting in an unknown concentration.

After the desired exposure period the glass flasks were opened and ventilated in a fume hood for at least one hour during which the glass vials of mites and beetles were removed for mortality assessment. Adult mortality in *N. rufipes* was determined by lack of motion when a beetle was actively disturbed with a probe within a ten-day post-treatment recovery period. The larval mortality of ham beetle was determined from the number that failed to reach the pupal stage within the time that all untreated larvae became pupae, and similarly the mortality of pupae was assessed from those that did not eclose to the adult stage. Eggs were checked daily for hatching after exposure until no further hatching was observed. Adequate diet was added into the vials to prevent cannibalism during the recovery periods. Mite mortality was assessed in a way similar to that used for ham beetles: failure of eggs to hatch and death followed by desiccation in the treated mobile stages. All the post-treatment recovery periods were in our laboratory incubator (Thermo Scientific, Buffalo, NY, USA) at 23 °C and 60%–70% RH, while vials of mites were further protected from stresses of variable humidity and excessive air circulation by placement in a glass desiccator chamber that contained a saturated NaCl solution to assure a constant RH of 70% for optimal survival.

### 2.3. Controlled Atmosphere Treatments

#### 2.3.1. CO_2_ Application and Quantification

Pressurized CO_2_ gas cylinders were custom-filled to the desired concentration (balanced with pure air) by the Linweld Gas Company (Linweld Lincoln, NE, USA) and delivered directly to us for use in these experiments. The separate cylinders were at concentrations of 10%, 20%, 40%, 60%, 80%, and 100% CO_2_. Experimental gas of a desired CO_2_ concentration was then introduced via Tygon^®^ tubing (Fisher Scientific) into the side-arm of an exposure flask on which the rubber stopper was partially sealed. The experimental CO_2_ was flushed through the flask for 60 s, after which the top stopper was tightly affixed and the sidearm tubing was pinched shut with a hose clamp. Quantitative gas chromatography-mass spectrometry, GC-MS, was performed with a Shimadzu GCMS QP5050A instrument (Shimadzu Scientific Instruments, Columbia, MD, USA) to measure the concentration of CO_2_ in each flask. The GC was equipped with a non-polar J and W Scientific (Folsom, CA, USA) DB-1 capillary column (30 m × 0.25 mm × 0.25 µm) in split mode with helium as the carrier gas and with a column flow rate of 1.3 mL/min. The injector temperature was 150 °C and the heated transfer line to the MS was set to 250 °C. The oven temperature was set isothermally to 100 °C. A standard curve was generated using the unaltered CO_2_ blend removed from the pressurized gas cylinder and held in a Tedlar^®^ bag (2 L) (CEL Scientific Corp, Santa Fe Springs, CA, USA), from which samples of different volume from 5–25 µL, all multiples of the known concentration were injected onto the GC-MS in which the MS was set to single ion mode to detect *m/z* of 44 for the molecular ion of CO_2_. Gas concentrations in jars were then determined from the area under the GC-MS peak of a 15 µL sample from a treated jar. The CO_2_ concentration in a given exposure flask was measured by GC-MS at the beginning of the exposure period and again at the end of the exposure period; the concentration assigned to that flask was the average of the starting and ending CO_2_ concentrations. The CO_2_ exposure periods were 0 (in air), 24, 48, 72, 96, 120, and 144 h for each of the concentrations. Exposure periods were designed to encompass a range of dose–responses, or presumed mortality at a given CO_2_ concentration for a given time, with the hope that a 100% kill could be achieved at the maximum concentration and time.

#### 2.3.2. Reduced O_2_ Treatment

Reduced O_2_ trials were conducted by placing ventilated vials with insects or mites into exposure flasks and then subjecting these to a vacuum to achieve a low pressure with concomitant reduction in O_2_ concentration. Rubber stoppers were fitted with dial-type pressure gauges (Grainger Inc., Lake Forest, IL, USA) that read units corresponding to mm Hg, and placed in the flask opening. The side-arm outlet of the flask was connected to a vacuum pump (General Electrical pump, Fairfield, CT, USA) via a vacuum hose equipped with a screw-type hose clamp. The air in the flasks was evacuated to the desired target pressure of 25 mm Hg, at which point the vacuum hose was clamped, the pump shut off, and the vacuum flasks placed in an incubator at 23 °C and 70% RH for the time periods needed for given experiments. Untreated control flasks were always set up at the same time and in the same way as treated flasks, but they were vented so they were at ambient pressure and maintained in the same chamber. The target pressure of 25 mm Hg (3.33 kPa), yielded an O_2_ concentration of 0.9%, was selected for this work as early studies on storage insects determine 25 mm Hg to be the lowest pressure that can be reliably attained under these conditions, and that the resulting low O_2_ concentration was known to give very good kill of all life stages [30]. Vacuum flasks were set up with three vials for each of the life stages for ham beetles and mites. Treatment flasks were held at 25 mm Hg for 24, 48, 72, 96, 120, and 144 h. Treated flasks with vials were vented after the desired exposure and held at 28 °C along with untreated vials of insects for the appropriate recovery period.

#### 2.3.3. Ozone Generation and Gas Analysis

A laboratory-scale ozone generator was generously provided by Adaptive Ozone Solutions Inc., Olathe, KS, USA (http://adaptiveozone.com/). O_3_ was generated from dry air using an electric discharge generator. The air flow rate was adjusted to 2 L/min. The generated ozone was deposited first into a 50 L plastic chamber for uniform distribution into the exposure flasks. The amount of generated ozone was regulated by adjusting the electric tension through a voltage regulator (dosing button). The exhaust of the ozone-treated chamber was connected to a Tygon tube. The generated ozone was first passed through an Erlenmeyer glass flask (1 L) containing water so that gas applied to the treatment was humidified before its release into the exposure chamber. Gas entering the exposure chambers was directly connected into the ozone monitoring instrument through a filter chamber for cleaning the generated ozone. The ozone concentration produced by the generator was measured with a continuous UV ozone monitor and the data were recorded and processed with associated software (IN-2000 LoCon Ozone Analyzer with Taltalk M5000 software, IN USA Inc., Needham, MA, USA), from which concentration for each chamber could be determined. Ozone toxicity was assessed following time-response bioassays in the exposure flasks. The time-mortality curves were established using increasing periods of exposure to ozone ranging from about 50 to 150 ppm. Preliminary tests were carried out to estimate the maximum and minimum exposure periods for the time-mortality bioassays and exposure intervals of 6, 12, 24, 36, and 48 h were then established. Ozone application was carried out under ambient temperature and relative humidity (27 °C and 70% RH), while the control chambers were placed in atmospheric air under the same conditions.

### 2.4. Statistical Analyses

Mortality data collected in each experiment were pooled together by replicate and sorted by life stages and treatments (exposure times). The means procedure (SAS Institute, Cary, NC, USA) was used to summarize average mortalities and corresponding standard error values of *N. rufipes* eggs, larvae, pupae, and adults, and for *T. putrescentiae* by the two life stage groups tested, eggs and mobile stages, and by different treatments. Raw data from these same dose-mortality bioassays were subjected to probit regression analysis (PROC PROBIT; SAS) for each experiment to predict the lethal exposure times for 50% and 99% mortality, the calculated fiducial limits around these estimates, the slopes and intercepts of the regression, and the goodness of fit of the regression to the actual data.

## 3. Results

CO_2_ concentrations in our exposure flasks never reached the levels of the source gas of known concentrations applied from the pre-mixed pressurized cylinders. These lower concentrations were most likely due to incomplete flushing of flasks with the experimental gas at the beginning of an experiment, which then resulted in dilution from the ambient air remaining in the flask. Thus, jars treated with 100% CO_2_ did not exceed 80% when measured, while those receiving 80% CO_2_ did not exceed 70% upon measurement. CO_2_ concentrations of 10%, 20%, and 40% resulted in very low mortality of all *N. rufipes* life stages for exposure up to 96 h, so these concentrations were not pursued further. The mortality responses of life stages of *N. rufipes* at different exposure times with the two highest CO_2_ concentrations tested are shown in Table 1, and results of probit regression analyses are in Table 2. These two CO_2_ concentrations caused substantial mortality for all life stages of *N. rufipes* compared to untreated controls at all five of the exposure times. At the longest exposure time of 144 h there was nearly 100% death achieved with 62.5% and 75.1% CO_2_ for all stages of ham beetle except larvae (Table 1). Adult *N. rufipes* were more susceptible to either 62.5% or 75.1% CO_2_ compared to the other stages treated as reflected in the lower LT_50_ values of 30.22 and 17.88 h, respectively (Table 2). LT_50_ values for both CO_2_ concentrations suggest that *N. rufipes* larvae are the most tolerant life stage followed by eggs, pupae, and adults (Table 2). It is quite striking that larvae of *N. rufipes* exposed either to 62.5% or 75.1% CO_2_ experienced no mortality at exposure times of 24, 48, and 72 h (Table 1), but at the higher exposure times of 96 and 144 h *N. rufipes* larvae had 90% or lower mortality, while eggs, pupae and adults had 100% mortality at 144 h. Estimated times for killing 99% of all the stages of *N. rufipes* at the highest CO_2_ concentration of 75.1%, which would be the minimum target mortality for many pest mitigation programs, was 437.7 h, with up to 1237 h at the 95% fiducial limit (Table 2).

The mortality responses of *T. putrescentiae* exposed to six concentrations of CO_2_ for different exposure periods are summarized in Table 3, and estimates for lethal exposure times are in Table 2. Mite bioassays showed that CO_2_ concentrations below 49.2% at exposure times less than 72 h were not effective against *T. putrescentiae*, particularly for mobile stages (Table 3). We achieved nearly 100% death of all mite stages following the treatment at 62.5% CO_2_ for 72 h and longer. The present findings also showed that the higher concentration of 75.1% did not achieve 100% mortality at exposure times less than 72 h for the mite stages treated (Table 3).

Mortality of *N. rufipes* and *T. putrescentiae* life stages exposed to low oxygen at 25 mm Hg pressure are shown in Table 4 and estimates for lethal exposure times are in Table 5. Mortality from low pressure occurred in a positive dose-response manner and 100% mortality of *N. rufipes* was achieved at the longest exposure times, especially for larvae and adults. Eggs and larvae of *N. rufipes* were the most tolerant to low pressure, followed by pupae and adults based on LT_50_ values (Table 5). Adults had 100% mortality at low pressure held for 120 h exposure. Eggs of *T. putrescentiae* appeared the most tolerant to low oxygen, while mobile stages were the more susceptible stages for this species (Table 5). The estimated exposure period for the LT_50_ mortality for mite eggs was 106.8 h, which was nearly three-fold higher than that estimated for mobile stages (Table 5). In addition, the complete mortality of mite eggs was not achieved at 144 h exposure in our experiment (Table 4).

Results for the mortality of life stages of *N. rufipes* exposed to different O_3_ concentrations are reported in Table 6 and Table 7. Both life stage and ozone concentration had a significant effect on ham beetle mortality (*p* < 0.01 in all cases). A 48-h exposure to 155 ppm O_3_, the highest concentration tested, resulted in 100% mortality of eggs, pupae, and adults, but we found that some ham beetle larvae were still alive after a 48 h exposure to each of the three O_3_ concentrations tested. Overall, eggs and pupae seemed more susceptible to O_3_ than were larvae and adults, based on comparisons of LC_50_ values. O_3_ treatment had no apparent effect on *N. rufipes* larvae at short exposures of 6 or 12 h to concentrations of 66 and 117 ppm.

Mortality of *T. putrescentiae* exposed to different concentration of O_3_ are summarized in Table 8 and Table 9. Toxicity data for ozone treatments indicated a difference in susceptibility between life stages of *T. putrescentiae* based on LC_50_ values. Mobile stages of *T. putrescentiae* were more susceptible to O_3_ exposure than eggs, since 100% mortality was easily achieved in mobile stages with 37 ppm O_3_ after just 12 h exposure (Table 8). However, the high O_3_ concentration of 155 ppm was needed to achieve mortality near 100% of *T. putrescentiae* under most exposure times. Probit analyses of O_3_ dose-response trials indicated that *T. putrescentiae* eggs needed 81.87 h exposure for 99% mortality at 155 ppm, which was several fold higher than exposure times estimated for mobile stage mites (Table 9).

## 4. Discussion

Controlled atmosphere (CA) applications have been the subject of numerous studies that have revealed them to be effective against arthropod pests of stored commodities and as promising candidates to replace MB [2,31]. The experiments reported here point to the potential for three different CA methods to control two very different arthropod pests of southern dry-cured hams, one being a widespread synanthropic mite pest of high-value durable foods and the other a clerid beetle adapted to feeding on dried meats. Below we interpret our results and discuss their implications for future research on these three CA methods and their potential for commercial applications.

Very little research has been reported on the toxic effect of CO_2_ against the red-legged ham beetle. However, use of CO_2_ to control pests has been conducted for several other species of stored product beetles. Our results indicated that *N. rufipes* adults were relatively susceptible to CO_2_ while the larvae were the most tolerant among life stages for both concentrations of CO_2_ that were tested (Table 1). It was expected that adult beetles would be the most susceptible life stage, and eggs and/or pupae would be the most tolerant stages. Tolerance to CAs, in general, is predicted to be a function of respiratory activity of the insect tested, such that the more inactive life stages, the eggs and pupae, would be more tolerant while the highly active stages, the larvae and adults, would be more susceptible. Tolerance of eggs and pupae to CO_2_ has been shown for several other stored product beetle species, including *L. serricorne* [32], *Tribolium* spp. [33], and *Sitophilus oryzae* [34]. Our estimates for the LT_99_ of ham beetles to CO_2_ suggest that a commercial exposure at either of the two concentrations tested for controlling all life stages would require more than 51 days at 23 °C, which would be impractical. *T. putrescentiae* mortality was achieved at both concentrations of CO_2_ at exposure times of either 96 or 144 h. These results are in agreement with the findings of other researchers working with CO_2_ as a control for different species of mites, such as *Acarus siro* [35], *A. farris* and *T. longior* [36], and *T. farinae* [37]. As for the ham beetles, we also expected eggs of *T. putrescentiae* to be more tolerant to CO_2_ than the mobile life stages, but comparison of LT values between the life stages groups were inconclusive. LT_99_ values estimated from our data would suggest unreasonably long exposure times to kill mites under the conditions we studied.

Exposure to low O_2_ atmosphere (approximately 1% O_2_), compared to controls under an ambient atmosphere (approximately 21% O_2_), resulted in all tested life stages of both *N. rufipes* and *T. putrescentiae* experiencing substantial mortality at 23 °C. Estimated LT_99_ values of several hundreds of hours suggest that eggs would be the most difficult to kill for both species. Despite small sample sizes in the current study, our results add to the literature on this topic by again showing that reduced pressure resulting in low O_2_ has the potential for relatively simple control of stored product pests [18]. It is likely that the low temperature of 23 °C we used in all our CA studies here resulted in estimates of exposure times to be much longer than they might have been under warmer conditions. Mbata and Phillips [30] reported that exposure times for killing all life stages of three stored product pests could be reduced by 50% with an increase of 5 °C in temperature. Practical application of low O_2_ vacuum to a building would be difficult due to poor gas-tightness. Using an inert gas, such as N_2_, to purge an ambient treatment environment and reduce O_2_ may be possible, but application and maintenance of enough N_2_ is highly problematic [3]. Vacuum can be applied directly, safely, and economically to certain commodities held within gas-tight coverings or chambers for required times [18]. We propose that mite- or beetle-infested country hams could be directly treated with vacuum, but more research would be needed toward that objective because negative effects on the ham aging process, as with other quality effects, need to be studied.

Our experiments with O_3_ showed very good levels of mortality within reasonable exposure times at the highest concentration when considered against results for CO_2_ and low O_2_. These results concur with other research pointing to the efficacy of O_3_ against various other stored-product pests [19,24,38,39,40]. Despite the numerous species of stored grain pests tested with ozone in these earlier papers, the work reported here is perhaps the first to demonstrate activity for ham pests. It is interesting to note in our work that eggs and pupae of *N. rufipes* were more susceptible to O_3_ than larvae and adults, which is opposite to what one might expect for other CAs, and also for chemical fumigants. Low O_2_, high CO_2_ and gaseous fumigants like methyl bromide or phosphine, cause mortality via some direct respiratory modes of action. It has been demonstrated that O_3_ is highly reactive and damages cell membranes of organisms by causing oxidative stress [9,41]. The mechanisms which could confer tolerance to O_3_ by some insect life stages could include: (1) the impermeable insect body covering might allow little, if any, O_3_ penetration; and (2) the ability to close spiracles for prolonged periods could be insect stage-related and account for failure of O_3_ to reach target tissues in effective concentrations. Eggs and pupae can have soft integument and lack functioning spiracles, so could be more easily damaged by O_3_ than other stages. It has been postulated that O_3_, like ionizing radiation, exerts its major oxidative effects on biological tissue via a free radical mechanism(s). Therefore, O_3_ has been considered a “radiomimetic” agent [9,41].

CA treatments are considered relatively safe for human applicators, general bystanders, the environment, and food being treated, relative to chemical fumigants and other pesticides. Although people and other non-target organisms confined inside a CA-treated space would be at great risk of death, the venting of a CA into surroundings from a treated area would likely be much less dangerous than the release of a chemical fumigant. Food quality is generally not known to suffer from CA treatments [42], and work on the effects of CAs on dry-cured hams determined no deleterious effects on flavor volatile composition or sensory panel acceptance [43]. Practical or cost-effective application of CAs for dry cured hams, as with any durable commodity, will be among the factors that limit the adoption of CA treatments. Application of any of the CAs studied here to a buildings or rooms with aging hams, which is the context under which the industry would apply chemical fumigants for control of ham mites and beetles, is unlikely to be done. Most all buildings lack optimum gas-tightness for most typical fumigations, so it follows that saturating a building with levels of CO_2_ tested here, even if more effective than we determined, would be terribly cost-prohibitive. Vacuum or low pressure simply cannot be applied to a building to achieve a low O_2_ controlled atmosphere. The application of N_2_ to displace O_2_ would also suffer the same pitfalls as application of any other gas in a leaky building, though the potential of re-circulation with N2 concentration should be investigated [14]. However, CO_2_ or low O_2_ applied to gas-tight chambers or coverings with infested hams could likely serve as suitable alternatives to methyl bromide fumigation for these infested products. Additionally, the application of CO_2_ under a positive pressure can be very effective and reduce exposure time much lower than those reported here [44]. O_3_ should be pursued further for potential to use in chambers with infested hams, but also for larger spaces in a flow-through design, or perhaps even as directed flushing of specific spaces known for pest harborage. CAs may not be direct and equivalent replacements for fumigation of dry-cured hams, as with many other post-harvest pest systems, but they have the potential to be developed further into new methods to complement existing pest management practices.

## 5. Conclusions

The research reported here provides important information for developing non-chemical controls against two serious pest species of the dried ham and related food industries that are in desperate need for substitutes to methyl bromide fumigation. Future work on high CO_2_ and low O_2_ for controlling *N. rufipes* and *T. putrescentiae* could be done at higher temperatures for improved activity and at a commercial-level scale to help in developing an effective treatment in practice. High CO_2_ and low O_2_ would definitely require gas-tight structures for effective treatments, and such structures, though potentially very expensive, could be cost-effective for controlling infestation on high-value specialty products like aged hams and cheeses. Low O_2_ with vacuum, or via replacement with an inert gas like N_2_, would represent a very safe procedure for applicators and bystanders. A vacuum treatment adds nothing harmful to a work area when a chamber is vented, and the release of N_2_ would have little to no impact. Results with O_3_ were very encouraging and should facilitate further work to evaluate application scenarios and efficacy for these and similar pests. Application of O_3_ to stored grain has revealed challenges with rapid loss of gas due to degradation upon contact with grain masses with high surface areas [39,40], which then reduces effective levels of O_3_ needed for direct contact with target insects for toxicity. It is possible that application of O_3_ to surfaces of whole hams with mites or ham beetles could greatly impact these pests in a short time, and the application of O_3_ onto local floors, walls, and other surfaces and fixtures in ham aging rooms could reduce pest populations with very low risk.

## Figures and Tables

**Table 1:** Mean % mortality (±SE) of *N. rufipes* life stages treated with two concentrations of carbon dioxide at five different exposure times.*

Stage	Time (h)	Air Control	62.5% CO_2_	75.1% CO_2_
Eggs	24	0	26.7 ± 3.3	26.7 ± 3.3
	48	3.3 ± 3.3	30.0 ± 5.8	36.7 ± 3.3
	72	0	50.0 ± 0	46.7 ± 6.7
	96	0	80.0 ± 10.0	100
	144	6.7 ± 3.3	100	100
Larvae *	96	0	72.1 ± 5.8	75.2 ± 3.3
	144	0	60.0 ± 5.8	90.0 ± 0
Pupae	24	3.3 ± 3.3	26.7 ± 14.6	26.7 ± 3.3
	48	0	40.0 ± 5.8	36.7 ± 3.3
	72	0	56.7 ± 3.3	46.7 ± 6.7
	96	10.0 ± 0	30.0 ± 10.1	16.7 ± 3.3
	144	13.3 ± 8.8	93.3 ± 3.3	100
Adults	24	13.3 ± 6.7	33.3 ± 6.7	66.7 ± 3.3
	48	3.3 ± 3.3	56.7 ± 3.3	83.3 ± 6.7
	72	0	66.7 ± 17.7	93.3 ± 6.7
	96	6.7 ± 6.7	86.7 ± 6.7	100
	144	0	100	100

* No mortality of larvae occurred at CO_2_ concentrations lower than 62.5% at exposure times less than 96 h.

**Table 2:** Probit analyses for the estimated mortality of *N. rufipes* and *T. putrescentiae* exposed to CO_2_ for different time periods at 23 °C.

CO_2_ % Mean ± SE	Stages	N	LT_50_ h (95% Fiducial Limits)	LT_99_ h (95% Fiducial Limits)	Slope SE	Intercept ± SE	χ^2^ (df) (*p*)
Beetles at 62.5%	Eggs	150	53.13 (43.62–63.03)	305.12 (202.69–643.02)	13.19 ± 0.87	0.24 ± 5.19	15.85 (df = 13) (*p* = 0.26)
	Larvae	150	110.49 (92.90–142.10)	275.38 (188.69–903.31)	10.23 ± 2.10	50.19 ± 8.74	25.88 (df = 13) (*p* < 0.02)
	Pupae	150	50.51 (39.61–61.33)	214.16 (149.44–424.77)	13.73 ± 0.84	−5.98 ± 3.90	13.28 (df = 13) (*p* = 0.43)
	Adults	150	30.22 (24.98–49.34)	209.58 (129.38–703.56)	11.65 ± 1.39	−4.59 ± 9.67	21.99 (df = 13) (*p* < 0.05)
Beetles at 75.1%	Eggs	150	51.34 (41.89–60.98)	298.11 (197.99–631.03)	13.34 ± 0.57	−2.96 ± 2.70	15.79 (df = 13) (*p* = 0.26)
	Larvae	150	95.50 (88.25–103.30)	161.37 (140.19–208.04)	9.13 ± 1.27	46.96 ± 6.60	13.76 (df = 13) (*p* = 0.39)
	Pupae	150	56.06 (44.83–68.18)	437.73 (259.24–1237.00)	14.94 ± 0.53	−6.42 ± 2.32	12.99 (df = 13) (*p* = 0.45)
	Adults	150	17.08 (6.57–25.11)	150.70 (95.46–493.28)	23.32 ± 5.24	−128.45 ± 46.64	9.967 (df = 13) (*p* = 0.67)
Mites at 49.2%	Eggs	170	19.61 (9.42–30.08)	116.72 (65.94–502.12)	8.69 ± 2.14	−3.56 ± 17.54	48.95 (df = 15) (*p* < 0.01)
	Mobile Stages	360	30.86 (18.83–44.95)	312.45 (158.82–1436.00)	5.69 ± 0.68	−4.33 ± 9.67	77.17 (df = 16) (*p* < 0.01)
Mites at 62.5%	Eggs	170	20.98 (12.98–29.03)	114.93 (72.86–289.09)	8.76 ± 2.03	−3.07 ± 16.46	29.95 (df = 15) (*p* < 0.01)
	Mobile Stages	360	20.13 (13.37–27.53)	141.89 (87.31–347.88)	5.06 ± 0.96	−8.20 ± 15.55	54.62 (df = 16) (*p* < 0.01)

**Table 3:** Mean % mortality (±SE) of *T. putrescentiae* eggs and mobile life stages treated with six concentrations of carbon dioxide at five different exposure times.

Time (h)	Stages Assayed in Treatment Chambers with a Given % Carbon Dioxide						
	Air Control	12.0%	19.3%	35.0%	49.2%	62.5%	75.1%
Eggs							
24	6.7 ± 6.7	6.7 ± 3.3	6.7 ± 3.3	53.3 ± 3.3	63.3 ± 8.8	53.3 ± 8.8	50.0 ± 17.3
48	10.0 ± 5.8	3.3 ± 3.3	13.3 ± 3.3	66.7 ± 8.	96.7 ± 3.3	93.3 ± 3.3	100
72	6.7 ± 3.3	6.7 ± 3.3	13.3 ± 8.8	83.3 ± 6.7	100	100	96.7 ± 3.3
96	3.3 ± 3.3	6.7 ± 3.3	6.7 ± 3.3	76.7 ± 8.8	90.0 ± 10.0	90.0 ± 5.8	na
144	5.0 ± 4.0	15.0 ± 4.1	25.0 ± 4.1	95.0 ± 4.1	100	100	na
Mobile Stages							
24	18.3 ± 3.3	23.2 ± 4.4	20.0 ± 7.7	18.3 ± 4.4	16.7 ± 1.7	25.0 ± 2.9	70.0 ± 2.9
48	15.0 ± 5.8	18.3 ± 4.4	23.3 ± 3.3	35.0 ± 5.8	43.3 ± 7.3	95.0 ± 2.9	83.3 ± 6.0
72	18.3 ± 4.4	18.3 ± 3.3	20.0 ± 2.9	41.7 ± 3.3	88.3 ± 1.7	95.0 ± 5.0	91.7 ± 3.3
94	25.0 ± 2.9	25.0 ± 2.9	15.0 ± 2.9	25.0 ± 2.9	98.3 ± 1.7	100	na
144	13.3 ± 4.4	11.7 ± 6.0	20.0 ± 5.0	36.7 ± 6.0	100	100	na

na = refers to data not collected for these exposures.

**Table 4:** Mean % mortality (±SE) of the life stages of *N. rufipes* and *T. putrescentiae* held at a low oxygen atmosphere created under a vacuum-mediated low atmospheric pressure of 25 mm Hg for six different exposure times.

Species/Stage	Exposure Times (h)						
	0	24	48	72	96	120	144
*N. rufipes*							
Eggs	7.3 ± 3.3	33.3 ± 3.3	36.6 ± 16.6	60.0 ± 16.6	63.3 ± 12.5	93.3 ± 7.5	na
Larvae	0	13.3 ± 6.6	10.0 ± 6.6	23.3 ± 1.3	86.7 ± 7.5	100	na
Pupa	0	46.7 ± 3.3	53.3 ± 12.5	73.3 ± 3.3	86.7 ± 5.0	93.3 ± 2.5	na
Adults	0.7 ± 0.1	53.3 ± 7.5	73.3 ± 11.3	93.3 ± 3.3	100	100	na
*T. putrescentiae*							
Eggs	3.3 ± 3.3	6.6 ± 3.3	10.0 ± 6.6	3.3 ± 3.3	15.1 ± 3.3	63.3 ± 3.3	96.3 ± 3.3
Mobile Stages	21.4 ± 3.3	36.7 ± 6.6	41.1 ± 5.0	96.0 ± 3.3	91.3 ± 6.6	96.0 ± 1.6	100

na = data not collected for these exposures.

**Table 5:** Probit analyses for the estimated mortality of *N. rufipes* and *T. putrescentiae* treated at 25 mm Hg to achieve a low O_2_ atmosphere for different times at 23 °C.

Life Stages	N	LT_50_ h (95% Fiducial Limits)	LT_99_ h (95% Fiducial Limits)	Slope ± SE	Intercept ± SE	χ^2^ (df) (*p*)
Beetle						
Eggs	150	50.11 (20.08–78.48)	621.68 (220.90–NA)	11.68 ± 1.17	−1.79 ± 5.37	33.72 (df = 13) (*p* < 0.01)
Larvae	150	68.41 (47.63–98.22)	230.69 (137.56–1740.00)	10.89 ± 0.91	10.59 ± 3.91	54.85 (df = 13) (*p* < 0.01)
Pupae	150	32.153 (19.15–42.16)	399.35 (214.07–1796.00)	10.79 ± 0.69	−2.87 ± 3.65	12.38 (df = 13) (*p* = 0.50)
Adults	150	25.04 (16.91–31.36)	123.28 (88.37–240.55)	9.10 ± 0.67	−2.53 ± 4.08	14.19 (df = 13) (*p* = 0.36)
Mites						
Eggs	180	106.80 (75.80–217.86)	383.79 (199.43–NA)	14.15 ± 1.67	16.45 ± 5.81	89.00 (df = 16) (*p* < 0.01)
Mobile Stages	360	36.71 (25.53–46.20)	184.24 (125.65–403.81)	6.82 ± 0.48	−24.64 ± 5.84	49.04 (df = 16) (*p* < 0.01)

**Table 6:** Mean % mortality (±SE) of *N. rufipes* life stages exposed to three concentrations of ozone for five different exposure times.

Stage	Exposure (h)	Ozone Concentrations		
		66 ppm	117 ppm	155 ppm
Eggs	0	6.7 ± 3.3	13.3 ± 3.3	3.3 ± 3.3
	6	26.7 ± 6.7	33.3 ± 8.8	13.3 ± 3.3
	12	36.7 ± 8.8	46.7 ± 8.8	20.0 ± 5.8
	24	100	96.7 ± 3.3	100
	36	100	100	100
	48	100	100	100
Larvae	0	0	0	0
	6	0	0	0
	12	0	0	10.0 ± 0
	24	16.7 ± 8.8	56.7 ± 8.8	73.3 ± 8.8
	36	0	66.7 ± 13.3	76.7 ± 3.3
	48	40.0 ± 5.8	66.7 ± 3.3	96.7 ± 3.3
Pupae	0	16.7 ± 8.8	20.0 ± 5.8	30.0 ± 5.8
	6	56.7 ± 12.0	50.0 ± 5.8	83.3 ± 16.7
	12	56.7 ± 3.3	50.0 ± 10.0	93.3 ± 5.8
	24	100	70.0 ± 15.3	96.7 ± 3.3
	36	100	63.3 ± 6.7	96.7 ± 3.3
	48	100	83.3 ± 3.3	100
Adults	0	3.3 ± 3.3	20.0 ± 5.8	0
	6	6.7 ± 3.3	20.0 ± 5.8	30.0 ± 5.8
	12	10.0 ± 0	43.3 ± 8.8	33.3 ± 3.3
	24	50.1 ± 10.0	46.7 ± 8.8	70.0 ± 15.3
	36	86.7 ± 13.4	66.7 ± 17.7	96.7 ± 3.3
	48	66.7 ± 14.6	76.7 ± 12.0	100

**Table 7:** Probit analyses for the estimated mortality of *N. rufipes* treated with ozone at different concentrations and exposure times; NA = fiducial limits not available.

Insect Stages	O_3_ Conc. (ppm)	N	LT_50_ (h) (95% FLs)	LT_99_ (h) (95% FLs)	Slope ± SE	Intercept ± SE	χ^2^ (df) (*p*)
Eggs	66	150	10.44 (8.71–12.31)	37.37 (27.65–62.43)	3.80 ± 0.46	−2.43 ± 3.41	16.06 (df = 13) (*p* = 0.24)
	117	150	9.44 (7.58–11.30)	40.69 (29.24–72.49)	4.21 ± 0.51	−6.36 ± 3.79	12.89 (df = 13) (*p* = 0.46)
	155	150	12.79 (11.02–14.86)	35.52 (27.57–53.81)	3.43 ± 0.41	1.76 ± 2.94	18.62 (df = 13) (*p* = 0.14)
Larvae	66	150	70.80 (47.49−2379.0)	404.37 (130.20–NA)	7.22 ± 1.89	14.18 ± 3.54	19.83 (df = 13) (*p* = 0.09)
	117	150	29.34 (24.98–34.50)	122.39 (84.27–247.34)	4.49 ± 0.59	6.79 ± 2.74	17.20 (df = 13) (*p* = 0.19)
	155	150	21.11 (18.05–24.16)	65.86 (51.59–98.22)	3.89 ± 0.35	4.33 ± 2.06	10.57 (df = 13) (*p* = 0.64)
Pupae	66	150	6.70 (4.54–8.51)	40.35 (26.90–92.01)	4.39 ± 0.69	−10.49 ± 5.43	15.36 (df = 13) (*p* = 0.28)
	117	150	7.97 (0.84–14.07)	na	5.46 ± 1.18	−9.66 ± 7.18	14.22 (df = 13) (*p* = 0.36)
	155	150	1.25 (na)	50.88 (NA)	4.03 ± 1.22	−12.59 ± 10.68	20.09 (*p* = 0.09)
Adults	66	150	24.57 (17.70–34.63)	162.93 (84.50–900.09)	4.08 ± 0.65	5.80 ± 3.30	26.60 (df = 13) (*p* = 0.01)
	117	150	19.42 (13.70–27.07)	585.33 (207.85–7204.0)	5.15 ± 0.98	−2.44 ± 5.15	19.58 (df = 13) (*p* = 0.11)
	155	150	12.32 (8.58–16.20)	79.27 (47.41–239.51)	4.16 ± 0.38	−1.87 ± 2.59	20.94 (df = 13) (*p* = 0.07)

na = data not available as the value could not be calculated.

**Table 8:** Mean % mortality (±SE) of *T. putrescentiae* life stages exposed to two concentrations of ozone for five different exposure times.

Stage	Exposure (h)	Ozone Concentrations	
		37 ppm	155 ppm
Eggs	0	6.7 ± 3.3	5.3 ± 4.3
	6	3.3 ± 3.3	73.3 ± 12.0
	12	46.7 ± 3.3	86.7 ± 3.3
	24	50.0 ± 11.6	93.3 ± 3.3
	36	83.3 ± 3.3	93.3 ± 3.3
	46	96.7 ± 3.3	100
Mobile Stages	0	3.7 ± 2.2	na
	6	85.0 ± 5.0	na
	12	100	na
	24	100	na
	36	100	na
	48	100	na

na = data not collected at 155 ppm, as 100% mortality was observed at 66 ppm.

**Table 9:** Probit analyses of the mortality for *T. putrescentiae* treated with ozone at different concentrations and exposures.

Mite Stages	O_3_ Conc. (ppm)	N	LT_50_ h (95% Fiducial Limits)	LT_99_ h (95% Fiducial Limits)	Slope ± SE	Intercept ± SE	χ^2^ (df) (*p*)
Eggs	37	150	17.42 (14.28–20.79)	96.72 (66.54–181.47)	4.64 ± 0.26	−2.17 ± 1.17	15.44 (df = 13) (*p* = 0.28
Eggs	155	150	2.31 (0.15–4.91)	81.87 (38.24–1294.00)	3.14 ± 0.37	−2.15 ± 2.29	12.85 (df = 13) (*p* = 0.46)
Mobile Stages	37	150	5.33 (na)	6.96 (na)	1.39 ± 0.18	−1.44 ± 2.46	2.35 (df = 13) (*p* = 0.99)

na = data not available as the value could not be calculated.

## Abbreviations

The following abbreviations are used in this manuscript:

CA: controlled atmosphere
MB: methyl bromide
GC-MS: gas chromatography-mass spectrometry
LT: lethal time
